# Human Adipose-Derived Hydrogel Characterization Based on *In Vitro* ASC Biocompatibility and Differentiation

**DOI:** 10.1155/2019/9276398

**Published:** 2019-12-27

**Authors:** Omair A. Mohiuddin, Benjamen T. O'Donnell, J. Nicholas Poche, Rida Iftikhar, Rachel M. Wise, Jessica M. Motherwell, Brett Campbell, Suzana D. Savkovic, Bruce A. Bunnell, Daniel J. Hayes, Jeffrey M. Gimble

**Affiliations:** ^1^Center for Stem Cell Research and Regenerative Medicine, Tulane University School of Medicine, New Orleans, LA, USA; ^2^Department of Pharmacology, Tulane University School of Medicine, New Orleans, LA, USA; ^3^School of Medicine, Louisiana State University, New Orleans, LA, USA; ^4^Department of Pathology and Laboratory Medicine, Tulane University School of Medicine, New Orleans, LA, USA; ^5^Neuroscience Program, Brain Institute, Tulane University, USA; ^6^School of Medicine, Tulane University, New Orleans, LA, USA; ^7^Department of Biomedical Engineering, Pennsylvania State University, State College, PA, USA; ^8^LaCell LLC and Obatala Sciences Inc., New Orleans, LA, USA

## Abstract

Hydrogels serve as three-dimensional scaffolds whose composition can be customized to allow attachment and proliferation of several different cell types. Extracellular matrix-derived hydrogels are considered close replicates of the tissue microenvironment. They can serve as scaffolds for *in vitro* tissue engineering and are a useful tool to study cell-scaffold interaction. The aim of the present study was to analyze the effect of adipose-derived stromal/stem cells (ASCs) and decellularized adipose tissue-derived (DAT) hydrogel interaction on ASC morphology, proliferation, differentiation, and DAT hydrogel microstructure. First, the ASCs were characterized using flow cytometry, adipogenic/osteogenic differentiation, colony-forming unit fibroblast assay and doubling time. The viability and proliferation assays showed that ASCs seeded in DAT hydrogel at different concentrations and cultured for 21 days remained viable and displayed proliferation. ASCs were seeded on DAT hydrogel and cultured in stromal, adipogenic, or osteogenic media for 14 or 28 days. The analysis of adipogenic differentiation demonstrated the upregulation of adipogenic marker genes and accumulation of oil droplets in the cells. Osteogenic differentiation demonstrated the upregulation of osteogenic marker genes and mineral deposition in the DAT hydrogel. The analysis of DAT hydrogel fiber metrics revealed that ASC seeding, and differentiation altered both the diameter and arrangement of fibers in the matrix. Matrix metalloproteinase-2 (MMP-2) activity was assessed to determine the possible mechanism for DAT hydrogel remodeling. MMP-2 activity was observed in all ASC seeded samples, with the osteogenic samples displaying the highest MMP-2 activity. These findings indicate that DAT hydrogel is a cytocompatible scaffold that supports the adipogenic and osteogenic differentiation of ASCs. Furthermore, the attachment of ASCs and differentiation along adipogenic and osteogenic lineages remodels the microstructure of DAT hydrogel.

## 1. Introduction

Human adipose-derived stromal/stem cells (ASCs) are extracted from adipose tissue by collagenase digestion [1]. ASCs are multipotent cells, characterized by their ability to differentiate into mesenchymal lineage cells, including adipocytes, osteoblasts, and chondrocytes when cultured in specific media formulations [2, 3]. *In vitro* ASC culture experiments have been conventionally performed as a monolayer of cells grown on two-dimensional (2D) culture plates [4]. However, the simplicity of 2D cell culture systems presents an inherent flaw that they do not mimic the complex *in vivo* physiological microenvironment [4, 5]. In an effort to recapitulate *in vivo* conditions, recent research efforts have focused on culturing ASCs in three-dimensional (3D) systems like spheroids and hydrogels [5–7].

Hydrogels are versatile 3D scaffolds whose stiffness and composition can be customized to allow attachment and proliferation of a wide range of cell types [8]. Hydrogels can be produced from natural or synthetic polymers and tissue extracellular matrix ECM [5, 9]. Hydrogels are composed of at least 30% water by weight which ensures an adequate supply of nutrients to all cells and removal of cellular waste products [5, 10]. ECM hydrogels comprised of native tissue proteins, and growth factors are the closest replicates of the tissue microenvironment [10]. Several ECM hydrogels have become commercially available, most of which are derived from tumor basement membrane (TBM) [10]. A hydrogel of decellularized adipose tissue (DAT) can serve as an alternative ECM for research, due to the abundant availability of source tissue [11, 12] and accumulating evidence of its cytocompatibility [13]. DAT hydrogel and its composites support *in vitro* ASC proliferation [14] and adipogenic differentiation [15–17] and have proven to be adipoinductive scaffolds [9, 18–20]. Additionally, intact DAT scaffolds have displayed positive outcomes in wound healing [21] and nerve repair [22].

ECM-based hydrogels allow attachment of cells naturally due to the presence of collagens [23]. Cell attachment alters the ECM hydrogel architecture by contraction and reorganization of collagen fibrils. Moreover, matrix metalloproteinases (MMP) released by cells digest ECM proteins and remodel the scaffold [23, 24]. MMPs are a family of enzymes that are involved in morphogenesis, tissue repair, cell migration, and angiogenesis [25]. MMP-2 and MMP-9 digest collagen I, collagen IV, and other ECM proteins, which results in cell migration and ECM remodeling [25–27]. While MMP-2 is constitutively active in most cell types, MMP-9 activity is mainly observed in leukocytes [25, 27]. MMP-2 expression has been observed in ASCs, and its upregulation has been found to be associated with increased migration of ASCs and chondrogenic differentiation [27–29].

The aim of the present study was to analyze the effect of ASC-DAT hydrogel interaction on ASC morphology, proliferation, differentiation, and hydrogel microstructure. We hypothesized that ASCs can undergo adipogenic and osteogenic differentiation in DAT hydrogel, which could potentially remodel the hydrogel's microstructure via subsequent MMP expression.

## 2. Materials and Methods

### 2.1. Reagents

All reagents were purchased from Sigma-Aldrich (St. Louis, MO) or Thermo Fisher Scientific (Waltham, MA) unless otherwise stated.

### 2.2. Adipose-Derived Stromal/Stem Cell Characterization

Human ASCs (*n* = 3 donors) were received from LaCell LLC (New Orleans, LA). Pooled ASCs (Passage 2) were characterized using flow cytometry, differentiation (adipogenic and osteogenic), colony-forming unit-fibroblast (CFU-F) assay, and doubling time.

#### 2.2.1. Flow Cytometry

ASCs were labelled with fluorophore-conjugated antibodies against cell markers including CD3, CD14, CD31, CD45, CD73, CD90, and CD105. Labeled ASCs were analyzed by flow cytometry (Gallios, Beckman Coulter; Brea, CA) according to a published method [30]. Immunophenotypic markers were selected based on the joint recommendation of the International Federation for Adipose Therapeutics and Science (IFATS) and the International Society of Cellular Therapy (ISCT) published in 2013 [3].

#### 2.2.2. Differentiation

ASCs were grown on cell culture plates up to 80% confluence in stromal media. Thereafter, they were cultured in adipogenic media (AdipoQual™; LaCell LLC; New Orleans, LA; *n* = 3), and osteogenic media (10 nM dexamethasone, 20 mM *β*-glycerophosphate, and 50 *μ*M L-ascorbic acid; *n* = 3) for 28 days, with the media being replaced every 2-3 days. Adipogenic and osteogenic differentiations were assessed by Oil Red O and Alizarin Red staining, respectively, according to previously described methods [31, 32].

#### 2.2.3. CFU-F Assay

ASCs (*n* = 3) were plated on 10 cm cell culture plates at a seeding density of 100 cells per plate. The cells were cultured for 2 weeks, after which the plates were washed 3 times with phosphate-buffered saline (PBS) and stained with 3% crystal violet for 30 minutes. The plate was then washed with tap water, and ASC colonies (blue) larger than 2 mm in diameter were counted. CFU-F data are expressed as the number of colonies per 100 cells seeded [32].

#### 2.2.4. Proliferation and Doubling Time

ASCs were seeded in a 6-well plate at a seeding density of 1 × 10^4^ cells per well and cultured in stromal media. Every 24 hours, ASCs were detached using 0.25% trypsin/EDTA, stained with trypan blue, and live cells were counted. The procedure was repeated every 24 hours for 5 days. Cell doubling times were calculated using the following equation as described before [33, 34]:

(1) $$\begin{array}{c} \text{DT}=\frac{\text{CT}\times \ln2}{\ln\left(\text{Nf}/\text{Ni}\right)}, \end{array}$$

where DT is the doubling time, CT is the culture time, Nf is the final cell number, and Ni is the initial cell number.

### 2.3. Decellularized Adipose Tissue Hydrogel Preparation

Adipose tissue was obtained from LaCell LLC under an approved IRB protocol (Western IRB Study # 1138160, Puyallup WA) with written informed consent from donors (*n* = 3; BMI = 24.56 ± 2.52; age = 50 ± 5.7, mean ± standard deviation) undergoing elective abdominoplasty. Pooled adipose tissue from the three donors was decellularized and converted in to a prehydrogel (50 mg/mL) using the method described previously [35].

### 2.4. ASC Viability and Proliferation in DAT Hydrogel

ASCs were seeded in DAT hydrogel by first suspending the cells in prehydrogel at concentrations of 0.25 × 10^6^, 0.5 × 10^6^, 1 × 10^6^, and 2 × 10^6^ cells per mL (*n* = 4 for each concentration). Subsequently, the prehydrogel suspensions were pipetted on cell culture plates and incubated at 37°C for 30 minutes to form a stable composite hydrogel. The hydrogels were then submerged in stromal media, and ASC viability and proliferation were assessed over a 21-day period.

For viable cell imaging, the hydrogels were washed twice with PBS for 15 minutes, followed by incubation in calcein AM (10 *μ*M) for 30 minutes, and finally washed with PBS for 1 hour (all incubations were performed at 37°C). Viable cells were imaged using a Cytation 5 imaging reader (Calcein; 469/525 nm; BioTek Instruments Inc.; Winooski, VT). Alamar blue assay was used to quantitatively analyze cell viability at each time point based on the quantification of reduced alamar blue fluorescence intensity [36]. The hydrogels were submerged in alamar blue solution (1 : 10 dilution in fresh stromal media) overnight at 37°C. The supernatant (100 *μ*L) was then analyzed using Synergy HTX multimode reader (BioTek Instruments Inc.; Winooski, VT) at 540/600 nm [37, 38].

### 2.5. Adipogenic and Osteogenic Induction of ASCs in DAT Hydrogel

ASCs were seeded in DAT hydrogels at a concentration of 2 million cells per mL, using the method described in Section 2.4. ASC-seeded hydrogels (*n* = 6) were cultured in stromal media (control), adipogenic media, and osteogenic media for 14 or 28 days. The samples were harvested at day 14 or 28 postinduction and analyzed by quantitative reverse transcriptase polymerase chain reaction (qRT-PCR), histochemistry, scanning electron microscopy (SEM), and gelatin zymography.

### 2.6. Quantitative Reverse Transcriptase Polymerase Chain Reaction

The hydrogels were frozen with liquid nitrogen and milled using mortar and pestle. Total RNA was isolated from the powdered hydrogel using RNeasy® Mini Kit (Qiagen; Hilden, Germany). Subsequently, the mRNA was reverse transcribed using iScript™ cDNA Synthesis Kit (BioRad, Hercules, CA), followed by qRT-PCR using IQ™ SYBR® Green Super mix (BioRad, Hercules, CA). All qRT-PCR data are expressed as the relative mRNA expression (fold), which was calculated from the ΔΔCt values determined by normalizing the ΔCt values for differentiated samples to the controls (cultured in stromal media). Human primer sets were purchased from Integrated DNA Technologies (Coralville, IA, USA). The primer sequences are as follows (5′-3′): GAPDH forward: GGCCTCCAAGGAGTAAGACC, GAPDH reverse: TGGTACATGACAAGGTGCGG; adiponectin forward: AACATGCCCATTCGCTTTAC, adiponectin reverse: AGAGGCTGACCTTCACATCC; FABP4 forward: TGGGCCAGGAATTTGACGAA, FABP4 reverse: GCGAACTTCAGTCCAGGTCA; PPAR*γ* forward: AGGCGAGGGCGATCTTG, PPAR*γ* reverse: CCCATCATTAAGGAATTCATGTCATA; perilipin forward: ACAAGTTCAGTGAGGTAGC, perilipin reverse: CCTTGGTTGAGGAGACAG; collagen 1A1 (COL1A1) forward: CATGTTCAGCTTTGTGGACCTC, COL1A1 reverse: AGGTGATTGGTGGGATGTCTT; RUNX2 forward: TCTGACCGCCTCAGTGATTT, RUNX2 reverse: AAGGACTTGGTGCAGAGTTCA; alkaline phosphatase (ALP) forward: GCCGGAAATACATGTACCCCA, ALP reverse: GCTCTTCCAGGTGTCAACGA; osteonectin forward: TCTTCTGGGCTCAGTCAGGAT, osteonectin reverse: CTGGGATAGACCACTGGGCA; MMP-2 forward: CAAGGAGAGCTGCAACCTGT, MMP-2 reverse: CCGCATGGTCTCGATGGTAT.

### 2.7. Histochemistry

The hydrogels were fixed in 4% paraformaldehyde (PFA) and embedded in O.C.T. compound. Hydrogel sections (5 *μ*m) were von Kossa (ScyTek Lab, Logan, UT) stained, to assess mineralization of the scaffold in osteogenic samples [39]. For the analysis of adipogenic differentiation, hydrogels were fixed in 4% PFA, followed by BODIPY™ (2 *μ*M) and DAPI (300 nM) staining to visualize neutral lipid droplets and nuclei, respectively [40]. Image acquisition of von Kossa-stained slides was performed by Aperio ScanScope (Leica Biosystems; Wetzlar, Germany), while BODIPY™-stained (469/525 nm) and DAPI-stained (377/447 nm) hydrogels were imaged using the Cytation 5 imaging reader (BioTek Instruments Inc.; Winooski, VT).

### 2.8. Scanning Electron Microscopy

Samples were prepared for SEM by fixing the hydrogels in 4% PFA overnight, followed by dehydration in graded ethanol solutions. The samples were then dried using DCP1 critical point drying apparatus (Denton Vacuum Inc.; Moorestown, NJ) and sputter (Electron Microscopy Sciences; Hatfield, PA) coated with platinum for 4 minutes. The samples were imaged with FEI quanta 3D FEG FIB/SEM (5.0 KV). SEM images at 50,000x magnification were analyzed using ImageJ software (DiameterJ plugin) to determine the mean fiber diameter, pore size, and fiber intersections/*μ*m^2^ as described previously [41, 42]. Fiber morphology metrics observed in control, osteogenic, and adipogenic DAT hydrogel samples were compared to the DAT hydrogel (without cells).

### 2.9. Gelatin Zymography

MMP-2 and MMP-9 activities were analyzed by gelatin zymography using a modification of the methods described previously [29, 43, 44]. ASC-seeded hydrogels (*n* = 3) were cultured in stromal, adipogenic, and osteogenic media for 14 or 28 days. The hydrogels were harvested, then lysed in ice-cold NP-40 lysis buffer for 20 minutes and centrifuged (16,000 g; 20 minutes; 4°C). The supernatant was collected, and protein concentrations were determined using bicinchoninic acid assay (Pierce™ BCA protein assay kit; 23225). The samples were diluted to 4 *μ*g with 4x Laemmli buffer (1610747; Bio-Rad Labs, Hercules, CA) and run on the gel (10% polyacrylamide, 1.7 mg/mL gelatin) at 130 V for 120 minutes. Fetal bovine serum (FBS) was used as the positive control for MMP-2 and MMP-9. Following electrophoresis, the gels were washed twice in renaturing buffer (2.5% Triton X-100) for 30 minutes and incubated in substrate buffer (50 mM Tris HCl, 0.07% calcium chloride, 0.02% sodium azide) overnight. The gels were then stained with 0.5% Coomassie brilliant blue (30% ethanol, 10% acetic acid) for 60 minutes, followed by destaining (30% ethanol, 10% acetic acid) for 60 minutes. Consequently, white bands appeared against blue background indicating MMP-2 and MMP-9 activities. The gels were imaged using ImageQuant LAS 4000 (GE Healthcare; Chicago, IL), and the band areas were quantified by densitometry using ImageJ software.

### 2.10. Statistics

Statistical analyses were performed with Prism 5 software (GraphPad, San Diego, CA). All results are expressed as the mean ± standard deviation (SD). The data were analyzed using Mann-Whitney test or Wilcoxon matched-pairs signed rank test. The *p* values less than 0.05 were designated as statistically significant for all results.

## 3. Results

### 3.1. ASC Characterization Based on Immunophenotype, Differentiation, CFU-F Assay, and Doubling Time

**Figure 1:**
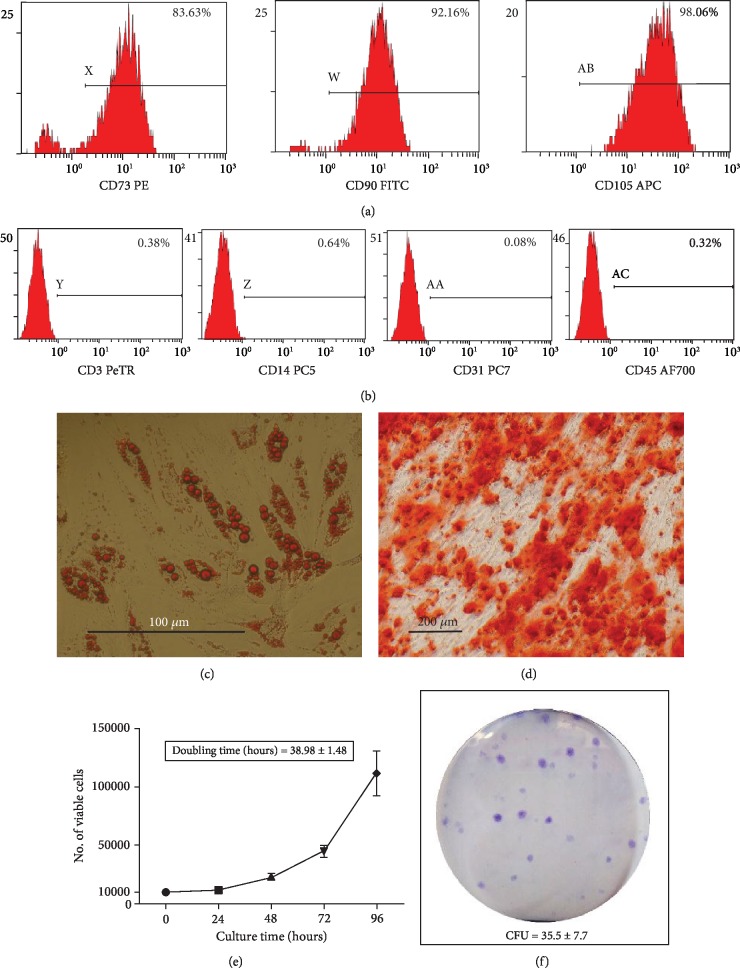
*ASC characterization based on immunophenotype, differentiation, doubling time, and CFU-F assay.* (a-b) Histograms display detection of surface antigen markers; (a) Positive markers CD73, CD90, and CD105; (b) Negative markers CD3, CD14, CD31, and CD45. (c) Representative image of Oil Red O stained cells; intracellular oil droplets (red) are visible in ASCs cultured in adipogenic media for 28 days (scale bar 100 *μ*m). (d) Representative image of Alizarin red stained cells; mineral deposition (red) is visible around ASCs cultured in osteogenic media for 28 days (scale bar 200 *μ*m). (e) Proliferation curve of ASCs from 0-96 hours, mean doubling time; 38.98 ± 1.48 hours. (f) Representative image of crystal violet stained ASC colonies; 35.5 ± 7.7 CFUs were observed per 100 cells seeded on cell culture plate and cultured in stromal media for 14 days. All displayed data are derived from 3-donor-pooled; Passage 2 ASCs.

Based on flow cytometry analysis, the ASC population was found to be positive for phenotypic markers CD73, CD90, and CD105 (Figure 1(a)), while negative for CD3, CD14, CD31, and CD45 (Figure 1(b)), consistent with the immunophenotype defined for ASC. Adipogenic differentiation of ASCs after 28 days of induction was confirmed by Oil Red O staining, where the cells stained positive for intracellular lipid vacuoles (Figure 1(c)). Osteogenic differentiation 28 days postinduction was confirmed by positive Alizarin Red staining, which indicated mineral deposition (Figure 1(d)). The ASC proliferation curve shows approximately a ten-fold increase in cell number from 0 to 96 hours (Figure 1(e)). ASC doubling times were calculated using cell count at each time point from 48 to 96 hours relative to 24 hours; the mean ASC doubling time was found to be 38.98 ± 1.48 hours ([Supplementary-material supplementary-material-1]). CFU-F assay showed that 35.5 ± 7.7 ASCs out of the 100 plated initially were able to form colonies (Figure 1(f)).

### 3.2. Effect of ASC Concentration on Viability and Proliferation in DAT Hydrogel

**Figure 2:**
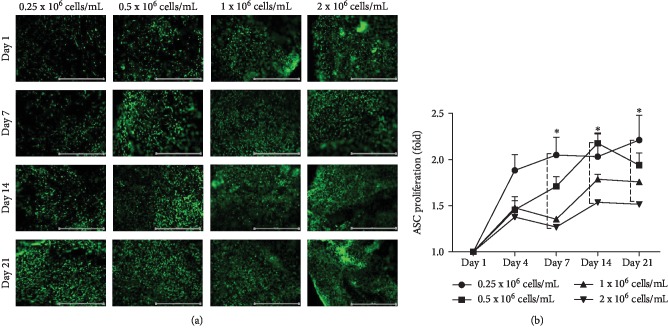
*Effect of ASC concentration on viability and proliferation in DAT hydrogel.* ASCs were seeded in the hydrogels at concentrations of 0.25 × 10^6^, 0.5 × 10^6^, 1 × 10^6^, and 2 × 10^6^ cells/mL and cultured in stromal media. Analysis of viability was performed on day 1 (1 day after seeding), day 4, day 7, day 14, and day 21. (a) Representative images of calcein AM stained viable cells (green); (b) ASC proliferation determined from fluorescence intensity (reduced alamar blue) for each time point relative to day 1 (fold). Data are expressed as mean (*n* = 4) ± SD; level of significance: (^∗^) *p* < 0.05; Scale bar 1000 *μ*m.

Calcein AM staining was used to visualize viable cells when seeded at different concentrations in DAT hydrogel and cultured over a 21-day period (Figure 2(a)). An increase in calcein-stained cells was qualitatively observed at all cell concentrations from day 1 to day 21 (Figure 2(a)). Fluorescence intensity of reduced alamar blue at each time point from day 4 to day 21 was normalized relative to the fluorescence intensity at day 1 to determine the fold increase in ASC viability, reported as a proliferation curve (Figure 2(b)). Time-dependent ASC proliferation was observed at all cell concentrations, with the fold increase at day 21 being 2.21 ± 0.47, 1.94 ± 0.22, 1.76 ± 0.027, and 1.516 ± 0.044 for 0.25 × 10^6^, 0.5 × 10^6^, 1 × 10^6^, and 2 × 10^6^ cells/mL, respectively. The highest proliferation increase was observed between days 1 and 4 at all cell concentrations except 0.5 × 10^6^ cells/mL which maintained consistent proliferation until day 14. Proliferation of ASCs seeded at 0.25 × 10^6^ cells/mL was found to be significantly higher than 2 × 10^6^ cells/mL at days 7 and 21. At 2 × 10^6^ cells/mL ASCs displayed lower proliferation than other cell concentrations (Figure 2(b)), yet viable ASCs were found to be distributed throughout the hydrogel (Figure 2(a)). Therefore, all subsequent ASC differentiation experiments were performed at this cell concentration.

### 3.3. Analysis of Adipogenic Differentiation of ASCs in DAT Hydrogel

**Figure 3:**
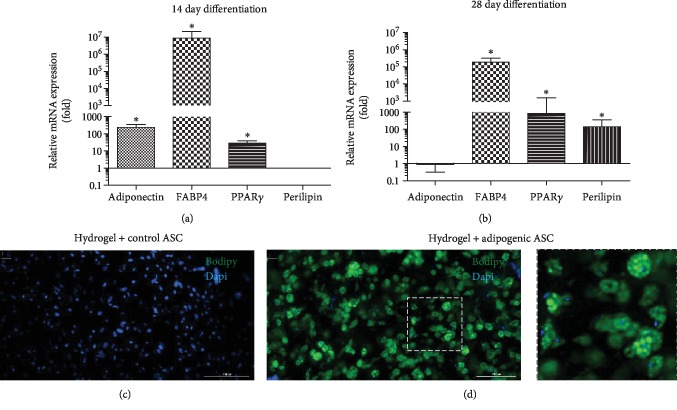
*Analysis of adipogenic differentiation of ASCs in DAT hydrogel.* ASC seeded DAT hydrogels (2 × 10^6^ cells/mL) were cultured in stromal or adipogenic media for 14 or 28 days and analyzed by qRT-PCR (a, b) and BODIPY staining (c, d). (a, b) Expression of adipogenic genes in adipogenically induced ASC samples after 14 (a) or 28 days (b) of culture is displayed as fold change relative to the control ASC samples. (c, d) Representative images of BODIPY/DAPI staining of 28-day Control ASC (c) and adipogenic ASC (d) seeded DAT hydrogels. Data are expressed as mean (*n* = 6) ± SD; level of significance: (^∗^) *p* < 0.05; Scale bar 200 *μ*m.

Adipogenic differentiation of ASCs in 3D cultures was analyzed by qRT-PCR and BODIPY™/DAPI staining. The qRT-PCR data revealed that in the 14-day samples, adipogenic genes including adiponectin, FABP4, and PPAR*γ* were significantly upregulated in the adipogenic samples (cultured in adipogenic differentiation media) relative to control samples (cultured in stromal media), while no perilipin expression was observed (Figure 3(a)). In the samples that were adipogenically induced for 28 days, the adiponectin expression level was found to be similar to the control samples, whereas FABP4, PPAR*γ*, and perilipin expression levels were significantly higher (Figure 3(b)). BODIPY™/DAPI staining of 28-day ASC-seeded DAT hydrogels displayed negative BODIPY™ signal in control samples, whereas positive BODIPY™ signal was observed in adipogenic samples confirming the presence of neutral oil droplets containing adipogenic cells (Figures 3(c) and 3(d)). The viability of control and adipogenic samples cultured for 28 days was confirmed by Calcein AM staining. The cells appeared viable in both samples but morphologically dissimilar, with the adipogenic cells transforming into a rounded morphology ([Supplementary-material supplementary-material-1]).

### 3.4. Analysis of Osteogenic Differentiation of ASCs in DAT Hydrogel

**Figure 4:**
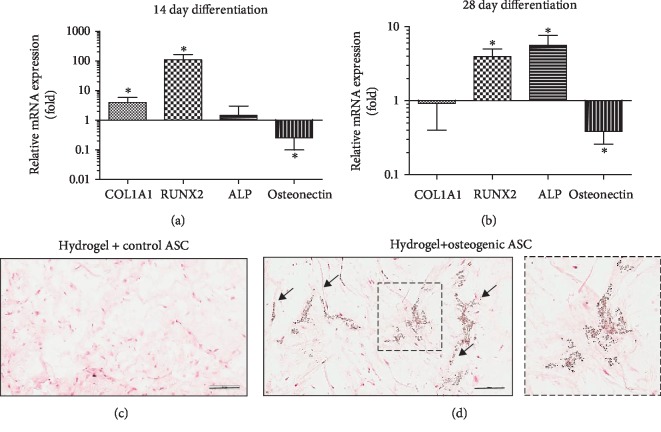
*Analysis of osteogenic differentiation of ASCs in DAT hydrogel.* ASC seeded DAT hydrogels (2 × 10^6^ cells/mL) were cultured in stromal or osteogenic media for 14 or 28 days and analyzed by qRT-PCR (a, b) and Von Kossa staining (c, d). (a, b) Expression of osteogenic genes in osteoegnically induced ASC samples after 14 (a) or 28 days (b) of culture is displayed as fold change relative to the control ASC samples. (c, d) Representative images of Von Kossa stained histological sections of Control ASC (c) and osteogenic ASC (d) seeded hydrogels. (d) Mineral deposits (brown) are indicated by arrows. Data are expressed as mean (*n* = 6) ± SD; level of significance: (^∗^) *p* < 0.05; Scale bar 200 *μ*m.

Osteogenic differentiation of ASCs in 3D cultures was assessed by qRT-PCR and von Kossa staining. The expression of the osteogenic genes COL1A1, RUNX2, ALP, and osteonectin was analyzed. The 14-day differentiation samples displayed significant upregulation of COL1A1 and RUNX2; ALP was not significantly upregulated, while osteonectin was found to be significantly downregulated relative to the control samples (Figure 4(a)). At the 28-day time point, RUNX2 and ALP were significantly upregulated and, like the 14-day time point, osteonectin was found to be significantly downregulated relative to the control samples (Figure 4(b)). von Kossa-stained sections (5 *μ*m) of 28-day samples provided evidence of mineral deposition in osteogenic samples, while no mineral deposits were observed in the control samples (Figures 4(c) and 4(d)). Osteogenic ASCs (differentiated for 28 days) were found to be viable and presented distinct morphological features in comparison to the adipogenic samples ([Supplementary-material supplementary-material-1]).

### 3.5. ASC and Hydrogel Fiber Morphology

**Figure 5:**
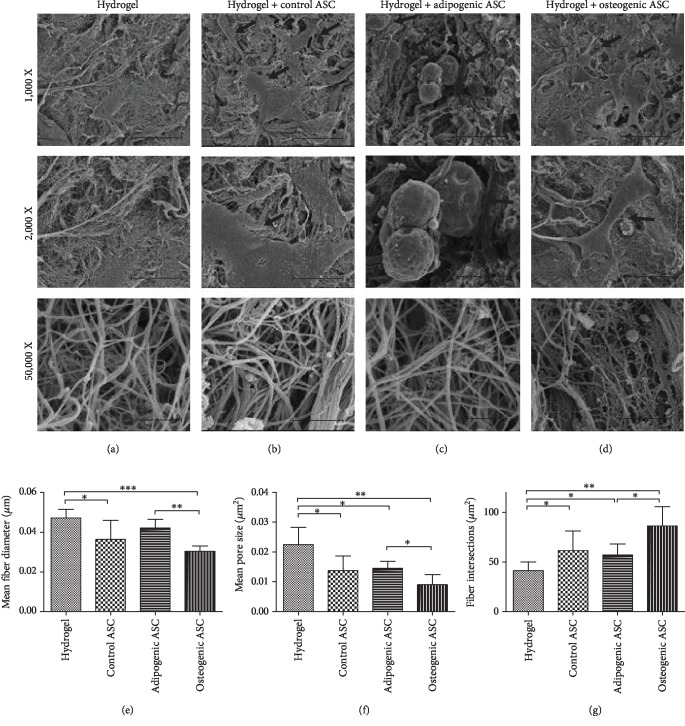
*Analysis of ASC and DAT hydrogel fiber morphology using images acquired by SEM.* (a-d) Representative SEM images of DAT hydrogel (no cells) (a), control ASC (b), adipogenic ASC (c), and osteogenic ASC (d) seeded DAT hydrogels are displayed at 3 magnifications; 1,000X (Arrows indicate cells; Scale bar 50 *μ*m), 2,000X (magnified to display cell morphology; Scale bar 20 *μ*m), and 50,000X (magnified to display fiber morphology; Scale bar 1 *μ*m). Fiber morphology metrics were measured from 50,000X images using ImageJ software (diamterJ plugin) to determine mean fiber diameter (e) mean pore size (f), and fiber intersections/ *μ*m^2^ (g). Data are expressed as mean (*n* = 6) ± SD; level of significance: (^∗^) *p* < 0.05; (^∗∗^) *p* < 0.01; (^∗∗∗^) *p* < 0.001.

Images acquired by SEM were used to qualitatively analyze ASC morphology (Figures 5(a)–5(d)) and for the quantitative analysis of fiber morphology metrics (Figures 5(e)–5(g)). The analysis of SEM images revealed that the adipogenic ASCs displayed a distinct morphology as compared to the control and osteogenic samples. The adipogenic cells appeared rounded, while in contrast the control and osteogenic ASCs appeared flattened and covered a greater surface area (Figures 5(b)–5(d)). The analysis of hydrogel fiber morphology (using images acquired at 50,000x magnification) displayed a significantly lower mean fiber diameter in control and osteogenic samples relative to the hydrogel (Figure 5(e)). The mean fiber diameter in adipogenic samples was not found to be significantly different from the hydrogel; however, it was significantly higher than the osteogenic samples (Figure 5(e)). All ASC-seeded samples exhibited lower mean pore size than the hydrogel alone, while the mean pore size of osteogenic samples was significantly lower than that of the adipogenic samples (Figure 5(f)). A significantly higher number of fiber intersections/*μ*m^2^ were observed in all ASC-seeded samples, with osteogenic samples displaying significantly more fiber intersections than the adipogenic samples (Figure 5(g)). Overall, the osteogenic samples appeared to have altered the hydrogel structure to a greater extent than the control and adipogenic samples.

### 3.6. Analysis of MMP-2 Expression in Control vs. Differentiated Cells

**Figure 6:**
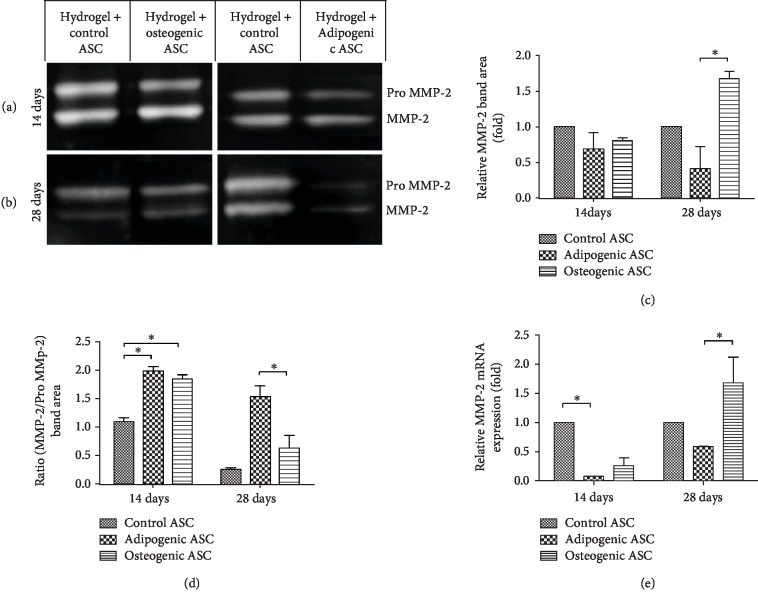
*Analysis of MMP-2 expression by gelatin zymography and qRT-PCR.* (a, b) Representative images of Pro MMP-2 and MMP-2 bands observed after gel electrophoresis of 14 (a) and 28 (b) day samples. (c) Pro MMP-2 and MMP-2 band area was determined by densitomteric analysis using ImageJ software, data are presented as fold change relative to the control samples. (d) Ratio of MMP-2 band area to Pro MMP-2 band area determined by densitometric analysis. (e) qRT-PCR analysis of MMP2 mRNA expression in control, adipogenic, and osteogenic samples are displayed as fold change relative to the control samples. Data are expressed as mean (*n* = 6) ± SD; level of significance: (^∗^) *p* < 0.05.

Gelatin zymography and q-RT-PCR were performed to analyze MMP-2 and MMP-9 expressions. Based on the results obtained from gelatin zymography, no MMP-9 expression was detected in any of the samples; however, a strong Pro MMP-2 and MMP-2 signal was detected ([Supplementary-material supplementary-material-1]). FBS was used as a positive control for MMP-2 and MMP-9 expressions. Two distinct bands were observed in the FBS lane, which corresponded to MMP-2 and MMP-9 based on the protein sizes determined from the ladder in lane 1 ([Supplementary-material supplementary-material-1]). Densitometric quantification of Pro MMP-2 and MMP-2 bands (Figures 6(a) and 6(b)) revealed that osteogenic samples displayed a significantly higher expression of MMP-2 relative to the adipogenic samples in 28-day samples (Figure 6(c)). The ratio of MMP-2/Pro MMP-2 was significantly higher in adipogenic and osteogenic samples relative to the control samples at 14-day time point, while the ratio was significantly lower in osteogenic samples relative to the adipogenic samples in 28-day samples (Figure 6(d)). Relative mRNA expression of MMP-2 displayed similar results to densitometric analysis of MMP-2 protein (Figure 6(e)). Significantly lower expression of MMP-2 was observed in adipogenic samples relative to the control in 14-day samples, while in the 28-day samples a significantly higher level of MMP-2 expression was observed in osteogenic samples relative to the adipogenic samples (Figure 6(e)).

## 4. Discussion

The present study shows that DAT hydrogel supports the attachment and proliferation of ASCs seeded at a wide range of concentrations. At each of the concentrations tested, viable ASCs were distributed across the hydrogel; however, the proliferation plateaued by day 21 in all samples. The ASC proliferation in DAT hydrogel was found to be inversely related to initial seeding concentration; i.e., at higher concentrations, the proliferation plateaued earlier, possibly due to earlier attainment of high confluence. As hypothesized, the ASCs were successfully induced to undergo adipogenic and osteogenic differentiation. Adipogenic differentiation was characterized by robust upregulation of adipogenic marker genes and a rounded cell morphology which closely resembled the morphology of adipocytes in their physiological form. Osteogenic ASCs displayed upregulation of osteogenic marker genes along with the deposition of minerals in DAT hydrogel, a phenomenon associated with active osteoblasts. Additionally, SEM-based evaluation revealed that all ASC-seeded DAT hydrogels had lower mean fiber diameter and pore size, while a higher number of fiber intersections per *μ*m^2^ was observed as compared to the unseeded DAT hydrogels. These data validated the hypothesis that ASCs could remodel the DAT hydrogel microstructure.

The current findings are consistent with several previous studies that have shown that ASCs remain viable and proliferate in DAT hydrogel [9, 14, 15, 17, 45]. Similarly, adipogenic differentiation of ASCs was observed in DAT hydrogel as reported in earlier publications. Tan et al. and Zhao et al. reported that DAT hydrogel serves as an adipoinductive scaffold [16, 18]. Moreover, DAT-composite hydrogels have also displayed biocompatibility and adipoinductive capabilities. Kayabolen et al. showed that DAT-silk fibroin hydrogels support vascularization and adipogenesis when seeded with adipoinduced ASCs and preendothelial cells [46]. Cheung et al. and Brown et al. reported that DAT-methacrylated chondroitin sulfate composite supports adipogenic differentiation of ASCs [15, 17].

PPAR*γ* is a key transcriptional activator in the early stages of adipogenic differentiation [47, 48] which was found to be upregulated at both time points postadipogenic induction. Additionally, the downstream targets of PPAR*γ* including FABP4, adiponectin, and perilipin [47] were upregulated at different time points. However, unlike earlier studies that had found DAT hydrogel to be adipoinductive for ASCs [16, 18], we did not observe spontaneous adipogenic differentiation of ASCs cultured only in stromal media. Moreover, the lipids in adipogenic ASCs appeared multilocular in contrast to the findings reported by Wu et al. [45], where unilocular lipid droplets were observed.

Osteogenic differentiation of ASCs in DAT hydrogel is a relatively novel finding. Although ASCs have a proven ability to undergo osteogenic differentiation when cultured in osteogenic media on a cell culture plate [1], very few if any studies have focused on the osteogenic differentiation of ASCs in DAT hydrogel or other tissue-derived scaffolds. However, ASCs and bone marrow-derived mesenchymal stem cells (BMSCs) seeded in synthetic hydrogels have been shown to undergo osteogenic differentiation and consequently deposit minerals in the 3D matrix [49–51]. Similarly, we observed osteogenic differentiation of ASCs with the upregulation of osteogenic marker genes and mineral deposition in DAT hydrogel. The upregulation of RUNX2, COL1A1, and ALP mRNA expression was consistent with previous findings showing that they are early to midstage markers of osteogenic differentiation [52]. Downregulation of osteonectin was observed at both time points which is in contrast with previous findings [53]. However, some studies suggest that undifferentiated ASCs seeded on collagen scaffolds express high osteonectin levels which are comparable to osteogenically induced ASCs from day 14 to day 28 [54, 55]. This could be the potential reason for the observation of the relatively low expression level of osteonectin in the current study.

Based on the analysis of SEM images, our data indicates that the attachment, proliferation, and differentiation of ASCs remodeled the microstructure of the DAT hydrogel. It has been postulated that the remodeling of the hydrogel fiber structure can be a result of cell attachment, migration, proteolytic activity, and/or ECM production [23]. DAT hydrogel remodeling was observed in all ASC-seeded samples, since they displayed a smaller mean pore size and a higher number of fiber intersections than unseeded DAT hydrogel. These alterations in DAT hydrogel fiber metrics were potentially a consequence of the attachment and migration of ASCs. Among the ASC-seeded DAT hydrogels, the fiber structure of osteogenically induced samples was found to be the most distinct in comparison to the unseeded DAT hydrogel. The potential mechanisms of DAT hydrogel remodeling induced by osteogenic ASCs include mineral deposition (as demonstrated by von Kossa staining and SEM images) and high proteolytic activity (induced by MMP-2).

The degradation of ECM proteins by MMPs allows the cells to remodel the surrounding microenvironment [23]. In the present study, we focused on MMP-2 expression to analyze the proteolytic activity of ASCs due to two primary reasons: (1) it is known through previous studies that ASCs express MMP-2 [27, 29], and (2) MMP-2 digests collagens I and IV [23, 26], which are two of the main structural components of DAT [11, 12, 14, 56].

In summary, the novel findings obtained from the study include that (a) DAT hydrogel supports osteogenic differentiation of ASCs, (b) ASC attachment and differentiation remodel the DAT hydrogel, and (c) MMP-2 is a potential regulator of DAT hydrogel remodeling.

The present study provides preliminary data to show that the interaction of ASCs and DAT hydrogel under different culture conditions results in uniquely remodeled scaffolds, further examination will be necessary to extend these initial observations. Although the MMP-2 expression profile provided an indication of the possible mechanism of scaffold remodeling, a more detailed account of overall MMP activity will be required. Furthermore, along with ECM degradation, the deposition of new ECM is an important aspect of scaffold remodeling. Since the current study limited its analysis to mineral deposition, additional evaluation of ECM synthesis, remodeling, and modification is warranted. Finally, previous studies have shown that better osteogenesis and osteoblast activity are observed on stiffer scaffolds [57, 58]. Therefore, chemical cross-linking strategies to enhance the DAT hydrogel's stiffness and subsequent osteoconductive potential will merit evaluation.

## 5. Conclusions

The present study shows that DAT hydrogel is a cytocompatible scaffold, which supports the adipogenic and osteogenic differentiation of ASCs. Furthermore, the attachment of ASCs and differentiation along adipogenic and osteogenic lineages remodels the fibrillar structure of DAT hydrogel. While the adipoinductive and adipoconductive nature of DAT hydrogel had been previously established in the literature, the osteogenic properties are relatively novel. Future studies of the DAT hydrogel will be needed to discriminate between possible osteoinductive and/or osteoconductive features of the scaffold. Together, the current findings confirm that DAT hydrogel can serve as a useful 3D model to study *in vitro* cell scaffold interactions and for tissue engineering applications.

## Data Availability

The raw/processed data required for these findings can be made available upon request by correspondence with Omair A. Mohiuddin (omohiudd@tulane.edu, omair.anwar@yahoo.com).

## Supplementary Materials

Supplementary Materials Table S1: ASC doubling times; based on viable cell count from 24 to 96 hours of cell culture. Figure S1: viability and morphology of ASCs seeded on DAT hydrogel. (A-C) Representative images of calcein AM-stained ASCs seeded on DAT hydrogel after 28 days of culture in stromal media (A), adipogenic media (B), and osteogenic media (C). Scale bar 1000 *μ*m. Figure S2: detection of MMP-2 using gelatin zymography. (A) Image of gelatin zymogram containing a standard protein ladder, positive control for MMP-2 and MMP-9, 14-day control, and adipogenic samples. (A-D) Bands for Pro MMP-2 and MMP-2 detected in 14- and 28-day samples cultured in stromal, adipogenic, and osteogenic samples.
