# Supplementary material for: Human Adipose-Derived Hydrogel Characterization Based on In Vitro ASC Biocompatibility and Differentiation
Source: Stem Cells Int. 2019 Dec 27;2019:9276398. doi: 10.1155/2019/9276398 (PMC7012213; doi:10.1155/2019/9276398)
Supplement: Supplementary Materials — Table S1: ASC doubling times; based on viable cell count from 24 to 96 hours of cell culture. Figure S1: viability and morphology of ASCs seeded on DAT hydrogel. (A-C) Representative images of calcein AM-stained ASCs seeded on DAT hydrogel after 28 days of culture in stromal media (A), adipogenic media (B), and osteogenic media (C). Scale bar 1000 μm. Figure S2: detection of MMP-2 using gelatin zymography. (A) Image of gelatin zymogram containing a standard protein ladder, positive control for MMP-2 and MMP-9, 14-day control, and adipogenic samples. (A-D) Bands for Pro MMP-2 and MMP-2 detected in 14- and 28-day samples cultured in stromal, adipogenic, and osteogenic samples. [file 9276398.f1.pdf]

Table S1: ASC doubling times; based on viable cell count from 24 to 96 hours of cell culture.

Culture time (hours)	No. of viable cells			Doubling time (hours)		
	Sample			Sample		
	1	2	3	1	2	3
24	11125	11125	13125			
48	25000	20000	25000	41.09	56.72	51.63
72	40000	45000	50000	39.00	35.71	37.31
96	90000	120000	125000	31.83	27.98	29.52
**Mean Doubling time**				**37.31**	**40.14**	**39.49**

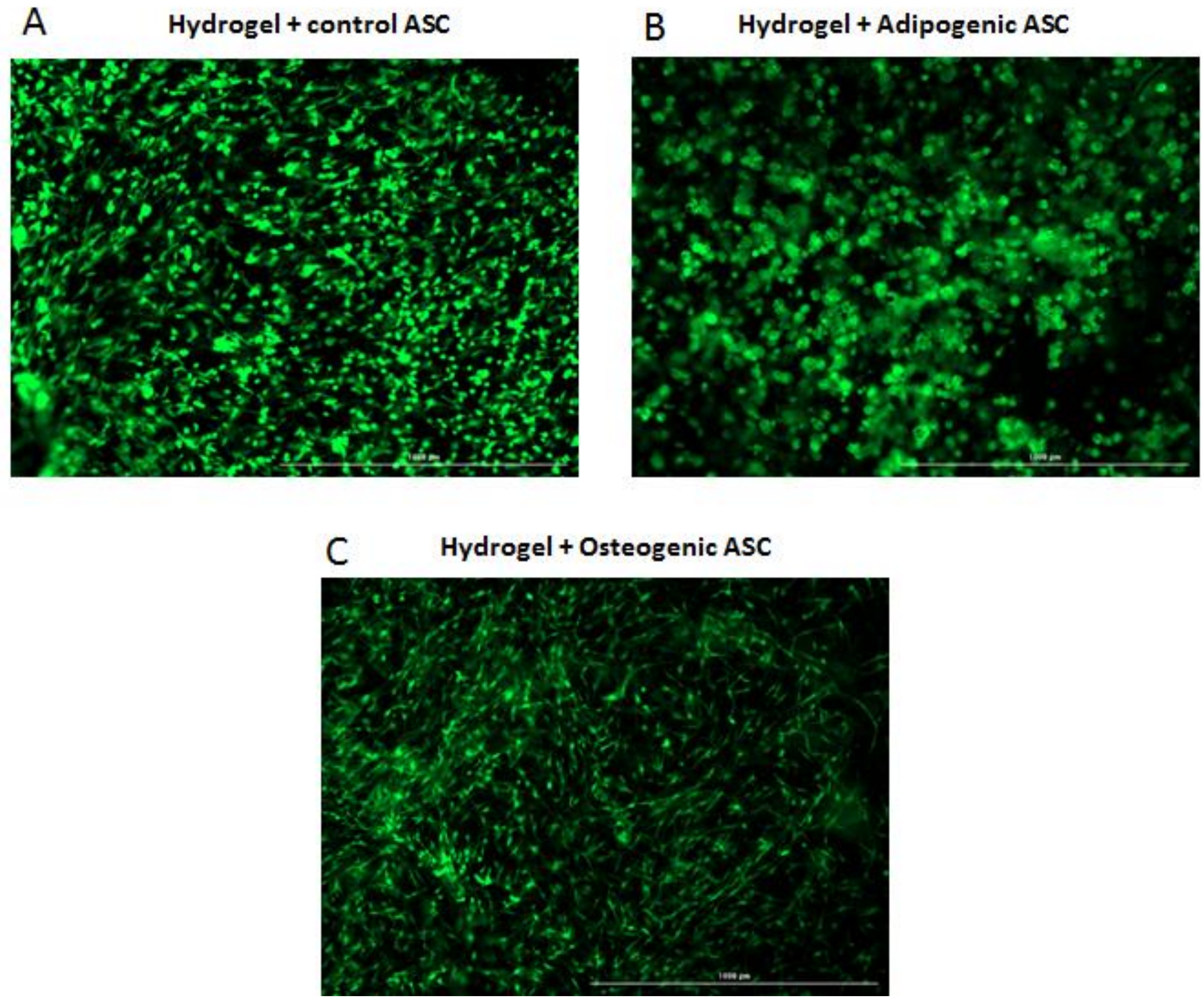

Figure S1: Viability and morphology of ASCs seeded on DAT hydrogel. (A-C) Representative images of calcein AM stained ASCs seeded on DAT hydrogel after 28 days of culture in stromal media (A), adipogenic media (B), and osteogenic media (C). Scale bar 1000 µm.

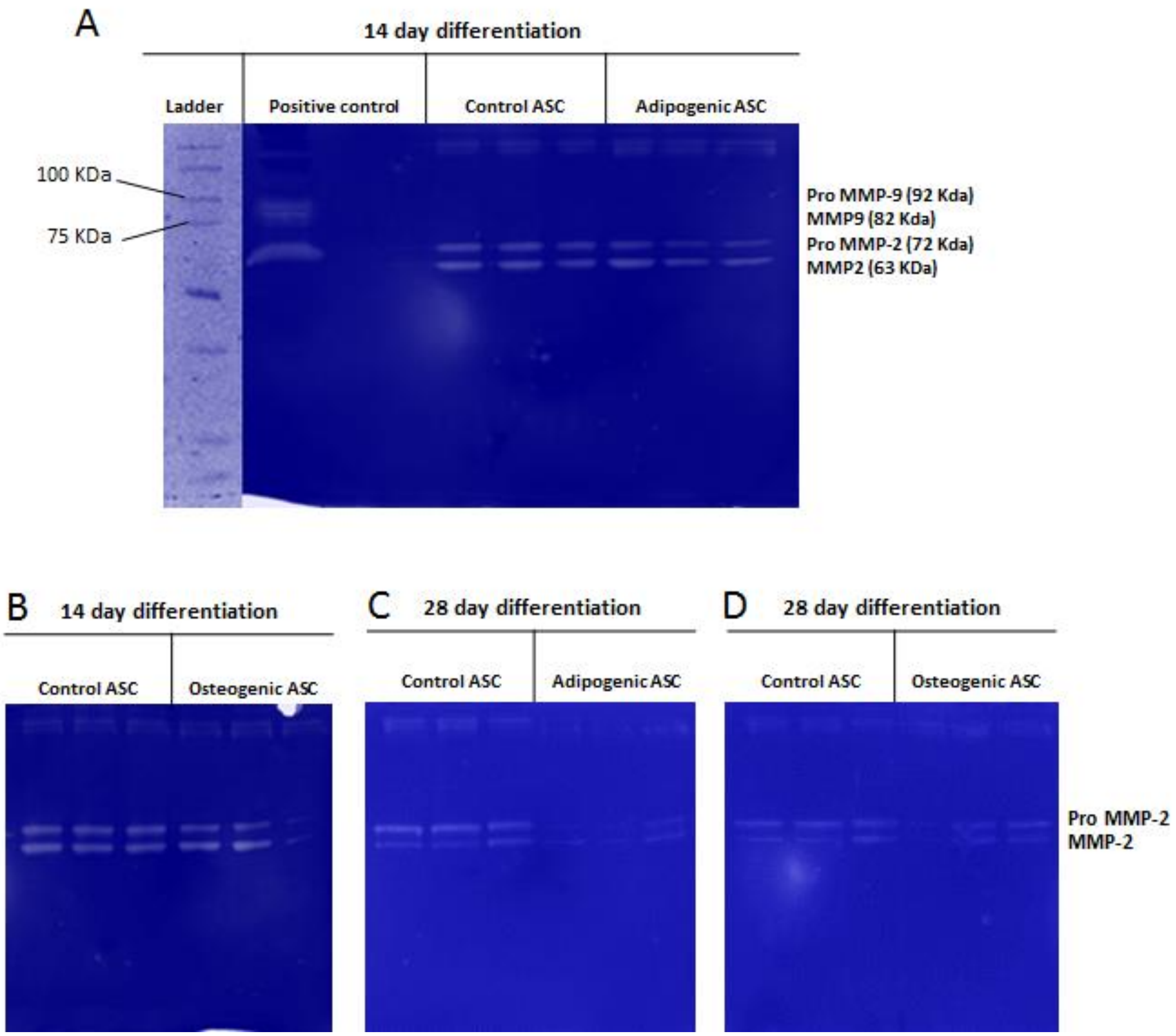

Figure S2: Detection of MMP-2 using gelatin zymography. (A) Image of gelatin zymogram containing a standard protein ladder, positive control for MMP-2 and MMP-9, 14-day control and adipogenic samples. (A-D) Bands for Pro MMP-2 and MMP-2 detected in 14- and 28- day samples cultured in stromal, adipogenic, and osteogenic samples.
